# American Cohort to Study HIV Acquisition Among Transgender Women in High-Risk Areas (The LITE Study): Protocol for a Multisite Prospective Cohort Study in the Eastern and Southern United States

**DOI:** 10.2196/14704

**Published:** 2019-10-03

**Authors:** Andrea L Wirtz, Tonia Poteat, Asa Radix, Keri N Althoff, Christopher M Cannon, Andrew J Wawrzyniak, Erin Cooney, Kenneth H Mayer, Chris Beyrer, Allan E Rodriguez, Sari L Reisner, American Cohort To Study HIV Acquisition Among Transgender Women (LITE)

**Affiliations:** 1 Center for Public Health and Human Rights Department of Epidemiology Johns Hopkins Bloomberg School of Public Health Baltimore, MD United States; 2 University of North Carolina School of Medicine Chapel Hill, NC United States; 3 Callen-Lorde Community Health Center New York, NY United States; 4 Department of Epidemiology, Johns Hopkins Bloomberg School of Public Health Baltimore, MD United States; 5 Whitman-Walker Health Washington, DC United States; 6 Department of Psychiatry and Behavioral Sciences, University of Miami Miller School of Medicine Miami, FL United States; 7 The Fenway Institute Boston, MA United States; 8 Division of Infectious Diseases, Department of Medicine, University of Miami Miller School of Medicine Miami, FL United States; 9 Division of General Pediatrics, Boston Children’s Hospital Boston, MA United States; 10 Pediatrics, Harvard Medical School Boston, MA United States; 11 Department of Epidemiology, Harvard TH Chan School of Public Health Boston, MA United States

**Keywords:** HIV, transgender persons, United States, cohort studies, HIV infection

## Abstract

**Background:**

In the United States, transgender women (TW) are disproportionately burdened by HIV infection. Cohort studies are needed to evaluate factors driving HIV acquisition among TW over time. These will require implementation strategies that are acceptable to the TW community and feasible to implement.

**Objective:**

This study aims to investigate the rate and correlates of HIV acquisition and other health outcomes among TW in eastern and southern United States.

**Methods:**

LITE is a multisite prospective cohort in 6 eastern and southern US cities, which will be followed across 24 months of technology-enhanced biobehavioral follow-up. Adult TW, regardless of HIV status, are recruited via convenience sampling (eg, peer referrals, social media, and dating apps). Participants are enrolled in a baseline study visit, complete a sociobehavioral survey, and test for HIV and sexually transmitted infections. Participants who are not living with HIV at baseline are offered enrollment into the cohort (N=1100); follow-up assessments occur quarterly.

**Results:**

Cohort assembly was informed by synchronous Web-based focus group discussions with TW (n=41) and by continuing engagement with community advisory board members from each site. Enrollment launched in March 2018. The study is underway in the Atlanta; Baltimore; Boston; Miami; New York City; and Washington, DC, metro areas. As of March 2019, 795 TW completed a baseline visit (mean age 35 years). The majority of the participants are racial/ethnic minorities, with 45% of the TW identifying as black and 28% of the TW identifying as Hispanic/Latinx. More than one-quarter (28%) of the TW are living with HIV infection (laboratory-confirmed). Online recruitment methods support engagement with TW, although peer referral and referral through trusted health facilities and organizations remain most effective.

**Conclusions:**

This study is responsive to increasing research interest in technology-enhanced methods for cohort research, particularly for hard-to-reach populations. Importantly, the diversity of literacy, technology use, and overall socioeconomic situations in this sample of TW highlights the need to leverage technology to permit a flexible, adaptive methodology that enhances engagement of potential participants living in marginalized contexts while still ensuring rigorous and sound study design.

**International Registered Report Identifier (IRRID):**

DERR1-10.2196/14704

## Introduction

### Background

Transgender women (TW) are one of the populations most affected by the HIV epidemic in the United States and internationally. A recent systematic review of global health burden in transgender populations further situates the HIV epidemic alongside other health conditions disproportionately facing TW [1]. Multiple biological, behavioral, and social risks for HIV infection among TW are driven by, and/or concomitant with, structural barriers that limit access to HIV prevention, testing, care, and health services [2]. In Baltimore; Washington, DC; Boston; New York City; Atlanta; Miami; and other US metropolitan areas, TW report histories of sexual assault and violence, homelessness, unemployment, substance use, and low health insurance coverage [3-12]. Substantial barriers have resulted in low access to HIV prevention, care, and other health services for TW [13-15]. Owing to these contextual vulnerabilities, as well as the acquisition risks of anal sex, which are common among TW, TW bear a higher and more disproportionate burden of HIV than the cisgender, heterosexual population. These factors increase the risk for HIV acquisition, resulting in an estimated 14.2% (95% CI 8.7%-22.2%) laboratory-confirmed HIV prevalence (meta-analysis) overall and as high as 44.2% and 25.8% weighted prevalence among black and Hispanic TW, respectively [16].

Disparities in HIV prevalence exist across races and ethnicity [5,17,18], with HIV prevalence among black TW being approximately twice that of other TW [19]. Self-reported HIV prevalence among young TW (aged 15-24 years) has ranged from 19% to 22%, highlighting early risk and onset of infection [8,20]. However, HIV prevalence by self-report may be an underestimate, given the low rates of HIV testing among TW [21]. Community-based HIV testing among transgender populations in New York City, Miami, and San Francisco (n=559) demonstrated a high (12%) prevalence of newly diagnosed HIV infection among TW participants [21]. Risk factors for new infections and unknown HIV infection included having a partner with unknown HIV status in the past year and having been tested more than 12 months ago [21]. Gender-based discrimination and prioritization of gender affirmation–related health care serve as barriers not only to HIV testing but also to engagement in HIV care and treatment [22-24]. Limited international cohorts that include small samples of TW from the United States suggest high HIV incidence, ranging from 2 to 4 cases per 100 person years (pys), among TW and an urgent need to develop and implement HIV prevention interventions that are both effective and acceptable to the TW population [25-27].

Promising empowerment-based and counseling interventions have demonstrated reductions in HIV risk behaviors among TW and their partners [28,29]. However, pre-exposure prophylaxis (PrEP) interventions, which have proven efficacious for other populations, have not shown efficacy for preventing HIV acquisition among TW, and benefits for this population remain unclear [25,30-32]. Many epidemiologic questions remain for TW, including the following: HIV incidence and risk parameters to inform HIV prevention trials; efficient and appropriate methods for recruitment and retention of participants; identification and characterization of HIV microepidemics; methods for secure dynamic data and real-time data capture among participants, given challenges with technology; and robust estimates of HIV care after seroconversion. Although TW have contributed to international and domestic HIV epidemiology and prevention research, they have not been adequately represented to produce scientific inferences for the population of TW alone.

Several challenges have affected the study of HIV among TW in the United States. First, the lack of transspecific marketing, misgendering during research (referring to TW participant with incorrect names/pronouns), and inclusion of TW among other key populations, such as men who have sex with men, have left a legacy of wariness and hesitation among TW as it relates to participation in HIV research. Despite historical participation in HIV research, TW participants have reported feeling that the successes of these efforts did not result in benefits for the transgender community and that HIV research was often neither specific nor acceptable to TW. Moreover, TW participants have expressed a need for research on other health priorities [33]. These findings highlight the need to assess other health issues within the context of HIV research, recognize and respond to TW priorities, and carefully market and promote HIV research with input from and dissemination to the community. Finally, although technology-based methods (typically necessitating mobile phones and internet access) have emerged as more efficient and cost-saving approaches to recruitment and data collection for HIV research, data have shown that TW frequently use social media, dating apps, and other internet-based media [34]. Our own experiences have found that mobile phone use is inconsistent among some TW because of loss/replacement of phones and temporary data plans. This suggests that thoughtful approaches to technology-enhanced methods for prospective study of TW are needed to ensure representation in research.

### Objectives

The objective of the American Cohort to Study HIV Acquisition among TW in High-Risk Areas—known locally and by participants as *Leading Innovation for TW’s Health and Empowerment* (*LITE*)—cohort study is to fill these critical scientific knowledge gaps in HIV prevention and care research and to translate findings into actionable interventions for TW in the United States. This study proposes to establish a multisite, racially/ethnically and culturally diverse prospective cohort of TW in eastern and southern United States to characterize HIV acquisition, risk factors for HIV infection, access to biobehavioral HIV prevention methods, and linkage to care for those who HIV seroconvert for the purposes of informing evidence-based and optimal HIV prevention interventions for this at-risk population. The specific aims of the study were as follows: (Aim 1) to determine the efficiency and acceptability of novel, technology-infused recruitment methods to enroll TW who are not living with HIV (here forward into a prospective cohort); (Aim 2) to describe the demographic, socioeconomic, behavioral, and physical and mental health profiles of HIV-uninfected TW in the first multisite longitudinal cohort of TW in eastern and southern United States; (Aim 3) to estimate HIV incidence, trends in incidence, and associated individual, social, and structural risk factors among TW in eastern and southern United States; and (Aim 4) to estimate the HIV Prevention Continuum (HIVPC) among HIV-uninfected participants and the HIV Care Continuum (HIVCC) among newly HIV-infected TW. The cohort will be supported by technology-enhanced recruitment and retention methods. This study will provide information needed about TW across 6 high-risk metropolitan areas (Boston; New York City; Baltimore City; Washington, DC; Atlanta; and Miami) in a region of the country where less is known about rates and unique risk factors for HIV acquisition among TW.

## Methods

### Overview

This is a multiple principal investigator (PI) study led by faculty from Johns Hopkins University (JHU; PI: Wirtz) and Boston Children’s Hospital (BCH), Harvard University (PI: Reisner). The study is supported by leading research institutions, spanning eastern and southern United States, including the following: JHU, Baltimore, Maryland; BCH, Harvard University, and The Fenway Institute at Fenway Health, Boston, Massachusetts; Callen-Lorde, New York City, New York; Emory University and Grady Memorial Hospital, Atlanta, Georgia; University of Miami, Miami, Florida; and Whitman-Walker Health, Washington, DC. The coinvestigators in this team specialize in HIV epidemiology among key populations, transgender health and clinical care, sociobehavioral sciences, management and analysis of longitudinal cohorts, and cross-sectional incidence estimation and laboratory methods. All partner organizations are affiliated with or are themselves clinical sites serving TW, and they have established strong relationships with local community-based organizations that specifically offer services for TW. These relationships continue to be nurtured to ensure the study is acceptable to the transgender community and is consistent with TW’s preferences in terms of study content, as well as to obtain valuable feedback on the study, which can support both recruitment and retention of study participants.

A virtual community advisory board (CAB) has been formed to facilitate research by serving as a mechanism for community consultation, and it will be engaged through every phase of the research activities. The CAB serves as a working partner and provides guidance to the research activities and consent processes; discusses concerns and protection of the community; and publicizes research activities, ancillary services, and results to the wider TW community [35,36]. The CAB is also comprised of transgender community leaders and those serving in transgender health care; it is representative of the diverse transgender communities residing in the high-risk locales enrolling this study, and it collaborates with investigators to foster meaningful research so that implementation is “with,” not “on,” TW [37]. The CAB took ownership by naming this project the LITE study.

### Conceptual Framework

This study draws on the *situated vulnerabilities* conceptual framework, which conceptualizes HIV infection risk and acquisition among TW, as recently described by Reisner et al [1]. Several conceptual models have been applied to transgender health (eg, social determinants and social ecological models [38,39], gender affirmation [40], gender minority stress [41,42], syndemic production [43,44], and health and human rights approaches) [45,46]. These models overlap in their shared recognition that multiple and intersecting levels of risk (biological, individual, interpersonal, and structural) shape the distribution of diseases in transgender populations. Thus, we conceptualized the multilevel factors that fuel HIV risk and acquisition among TW as “situated vulnerabilities” [23,47]. The vulnerabilities that TW face regarding HIV acquisition are both sex linked (biological per-act probability of HIV transmission in receptive anal sex) and gender linked (socially derived exposures such as stigma, survival sex, and gender affirmation). We focus on identifying the “situated vulnerabilities” driving HIV acquisition in the TW cohort. At the biological level, TW with anatomically male partners face a high HIV transmission probability via condomless anal sex with serodiscordant and viremic partners [48,49]. Coinfection with perigenital or perianal sexually transmitted infections (STIs) may also potentiate the acquisition and transmission of HIV [50,51]. Network-level risks include a high prevalence of HIV and limited awareness of HIV status within transgender-inclusive sexual networks [52-54]. Community-level stigma and structural-level discriminatory laws also contribute to the high burden of HIV by limiting the provision and uptake of services as well as by driving TW to engage in sex work for economic survival and gender affirmation [23,40,55].

### Design

This study focuses on the recruitment and development of an HIV-uninfected cohort as well as a cross-sectional HIV-infected comparison group. (Note: we recognize and prefer to refer to this group as “TW who are not living with HIV” to minimize stigmatization; however, for the purposes of this technical report, we utilize the term “HIV-uninfected” to refer to the diagnosis associated with study screening criteria.) TW, regardless of HIV status, are recruited via technology-based and nontechnology-based recruitment methods (Figure 1). HIV-uninfected TW who meet eligibility criteria are enrolled in the longitudinal cohort. TW who are recruited but are ineligible for the cohort on the basis of HIV infection are enrolled in a comparison cross-sectional sample and complete the same baseline survey and laboratory testing as the HIV-uninfected cohort.

HIV-uninfected TW are followed for a minimum of 24 months to estimate HIV incidence, trends, and risk factors for HIV acquisition (Figure 1). Following the facility-based enrollment visit, survey and laboratory data will be collected from cohort participants every 3 months using Web-based and facility-based visits. Web-based data collection utilizes an app-based self-administered survey and home HIV self-testing with photo validation of results. Any participant who seroconverts during the longitudinal study completes facility-based confirmation of HIV infection and is referred to affirming services for HIV care. Participants who seroconvert are followed for 1 additional study visit for the purposes of assessing prospective engagement in the HIVCC.

**Figure:**
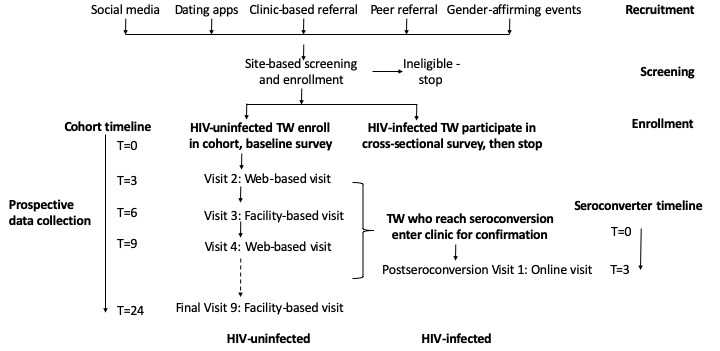
Study flow diagram. TW: transgender women.

### Sample

The overall study sample, inclusive of formative research, baseline, and cohort participants, is comprised of TW adults, aged ≥18 years, assigned male sex at birth but identifying as women or along the transfeminine spectrum. Gender identity is verified during enrollment by using the recommended 2-step method, which measures sex assigned at birth (step 1) and current gender identity (step 2) [56,57]. Enrollment in the cohort is restricted to HIV-uninfected TW, which is laboratory-confirmed at enrollment.

Cohort enrollment eligibility is determined during the baseline study visit. Eligibility includes a negative baseline HIV test result and at least one of the following risk factors for HIV acquisition (as HIV incidence is the primary outcome of this study): (1) aged 18 to 24 years; (2) screens positive for alcohol use disorder; (3) reports engaging in condomless anal and/or vaginal sex in the previous 12 months; (4) reports lifetime history of sex work; (5) reports unstable housing in the previous 12 months; (6) reports sharing needles to inject hormones, silicone, and/or drugs; (7) screens positive for drug use disorder; (8) reports a lifetime history of injection drug use; (9) reports a lifetime history of incarceration; (10) reports STI diagnosis in previous 3 months; (11) has a positive STI diagnosis at baseline; or (12) reports current or recent (last 12 months) PrEP use. Participants currently enrolled in an HIV prevention clinical trial are excluded.

Generalizability of study results is optimized using multiple study sites across eastern and southern United States. All 6 metropolitan areas are diverse racially, ethnically, and culturally; it is anticipated that the final study sample will be equally diverse, given the team’s past and ongoing research experience and the composition of TW served in clinical care services across sites. Findings may be less generalizable to rural areas; however, there is little known about the number of TW living in rural areas, and the use of online recruitment methods may facilitate participation of those living in more suburban and rural areas surrounding select metropolitan sites. Finally, the minimal use of facility-based data collection efforts during follow-up may help to protect against the Hawthorne effect that may compromise generalizability [58].

### Formative Research

Formative, qualitative research was conducted between August 2017 and January 2018 with members of the TW population to inform recruitment, marketing, and app development. Qualitative research utilized computer-mediated communication (CMC; “Web-based focus groups”) with focus groups comprising participants from across all 6 sites [59]. CMC provides multiple benefits in terms of reducing cost and barriers associated with finding an ideal time and physical space for participants to meet. Previous research comparing in-person with CMC focus groups has found that greater sharing of ideas occurs via CMC because of visual anonymity and that perceived distance of the internet stimulates group discussion and disclosure [60]. Our team has successfully used CMC with transgender participants previously [61].

Focus groups comprised 5 to 10 TW participants who met via video/teleconference at a mutually agreed upon time. Focus group participants were recruited using the same methods utilized to accrue the cohort and cross-sectional samples (see the Recruitment section), and maximum variation sampling was utilized to enroll participants across a wide range of race, ethnicity, age, geographic residence, and gender transition. Candidate participants were screened for eligibility (see the Sample section), regardless of HIV status, before enrollment, and they had the option to join by audio only, to use video, or to upload a photograph, depending on participant comfort and preference. Discussions were led by a facilitator, utilized audio communication to avoid delays associated with typed CMC, and followed best practices set forth for CMC qualitative research [60,62]. A professional Web-based meeting provider was used to maximize security, provide audio recording, and offer telephone options for participants without internet access. Semistructured discussion guides were used during data collection to identify preferred recruitment methods, assess acceptability of fingerprint or other verification methods for study participation, obtain input/feedback on study branding and app design, and identify concerns related to study activities.

Qualitative research was led by investigators from JHU and BCH (Wirtz and Reisner) using standardized methods for qualitative research with key populations [63,64]. Research was iterative in nature, bringing in results and new questions raised from earlier focus groups. An iterative, analytic approach followed Crabtree and Miller’s 5-step approach to qualitative interpretation: (1) describing, (2) organizing, (3) connecting, (4) corroborating, and (5) representing [65]. Regular debriefing meetings were held with focus group facilitators to identify preliminary findings and facilitate iterative data collection and analysis. An initial set of codes were developed based on the core domains described above. Qualitative codes were then applied to transcripts and discussion notes using NVivo qualitative analysis software (QSR International) by 2 independent analysts. New codes were developed as they emerged. Summaries were developed for major themes and distinctions across subgroups. Corroboration included discussing preliminary findings and implications for the cohort study with the CAB. Preliminary findings informed subsequent cohort methods, and final analytic findings have been submitted for publication [66].

### Recruitment

**Figure 2:**
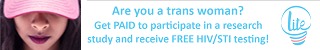
Electronic study flier.

Study recruitment utilizes a mixed format of technology- and nontechnology-infused recruitment methods. Technology-infused recruitment methods include the use of geosocial networking (GSN) apps frequented by TW, such as Grindr, Black Gay Chat, and Tinder, as well as social media, including Facebook and Reddit, all of which have been successfully used to recruit other populations [34,67-69]. Figure 2 displays a banner advertisement that was developed for the dating apps. GSN and social media recruitment are geotargeted to the 6 metropolitan areas and surrounding regions [68]. Study banners, messages, and broadcasts use images of TW, which reflected the racial, ethnic, and gender presentation diversity; provide a study phone number; and link to the Web page in English and Spanish. GSN sites also collect statistics such as the number of impressions, clicks, and time of each click that are used for analysis of recruitment efficiency.

Nontechnology-based recruitment methods include peer referrals and clinic-based referrals (excluding HIV-related services) as well as venue-based recruitment from gender-affirming community events. We used nontechnology-based methods in addition to technology-infused methods, given past research that showed that despite Web-based outreach, only 21% of transgender participants learned of the study via the internet, with the remainder recruited by a peer or support group [70]. Peer referrals follow a modified respondent-driven sampling (RDS) technique [71], allowing enrolled participants to recruit other TW peers to the study by using uniquely numbered electronic short message service (SMS)–based coupons for recruitment tracking purposes. Gender-affirming community events focus on conferences that introduce the community to a range of health professionals serving the transgender community (eg, endocrinology, surgical), offer networking and social support, as well as offer legal clinics and other referrals.

Conferences include but are not limited to the following: First Event, Boston [72]; Philadelphia Trans Health Conference, which is frequently accessed by community members from Baltimore-Washington, New York, Boston [73]; the Atlanta's Transgender Health and Education Alliance's Peach State Conference [74]; and Southern Comfort, Fort Lauderdale [75], accessed by TW in the Miami area. Partnerships with these events for recruitment allow the research team to acknowledge TW’s health and livelihood priorities and provide an opportunity to meet TW in a space that is gender affirming.

Participants are directed from study fliers, SMS text messages, or Web-based advertisements to the study Web page [76] where participants can complete an interest survey to be contacted by study staff or can directly identify the telephone number associated with their city and call to schedule an appointment. Candidates are required to attend a facility-based visit at the nearest study site for screening to verify eligibility (identify as TW based on a 2-step procedure [56,57], aged ≥18 years, and residing in/around 1 of the 6 metropolitan areas or surrounding regions) and complete baseline data collection. A unique identifier is created for participants to allow for secure access to follow-up surveys through the mobile app.

### Facility-Based and Electronic Study Visits

All TW who are recruited via the above-mentioned methods and meet eligibility criteria, regardless of HIV status, are asked to participate in a baseline survey, HIV and STI testing, and biospecimen collection. HIV-uninfected TW who meet cohort eligibility criteria are enrolled in the prospective cohort. TW who are recruited but are ineligible for the cohort on the basis of HIV infection are enrolled in a comparison cross-sectional sample and complete the same baseline survey and laboratory testing as the HIV-uninfected cohort.

Each cohort participant is followed for a minimum of 24 months (the duration anticipated for the funding duration, pending grant renewal). Survey and HIV self-testing results are collected from cohort participants every 3 months using a multimodal approach in which facility-based participation is required for baseline visit and 6-, 12-, and 24-month visits, whereas the rest are optional Web-based or facility-based visits (Figure 1). The required facility-based visits provide an opportunity to continue to build relationships between staff and participants, collect biologic specimens and test for STIs, and provide participants with additional HIV self-test kits that can be used at home in subsequent visits. Participants have the option to engage on the Web or in person at a site facility during the remaining study visits, given inconsistent ownership of or access to telephones and/or computers, variations in reading and technology literacy, and individual preferences for personal interactions with study staff. Participant choice for the mode of study visit is recorded. We elected to use this multimodal format to balance minimizing risk of bias (Hawthorne effect associated with facility-based data collection) with methods to optimize retention, recognizing that although TW have high rates of internet use [34], access can be inconsistent and reliance solely on Web-based data collection may threaten study retention.

**Figure 3:**
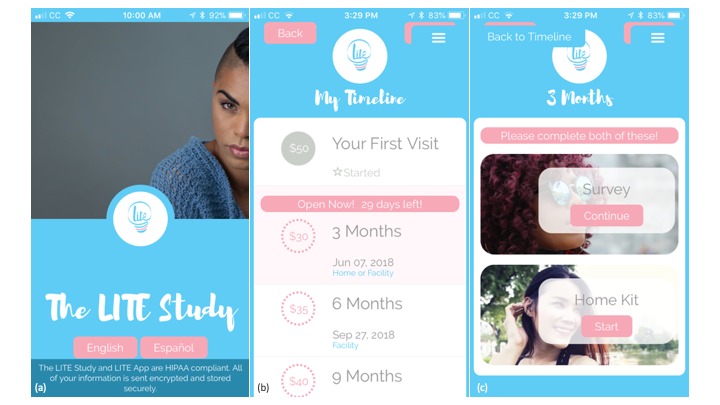
Leading Innovation for Transgender Women’s Health and Empowerment study app: (a) home screen, (b) study timeline, and (c) access to survey and entry page for home HIV self-test results.

Every 3 months, surveys will rotate length and format to maintain participant interest, although all surveys will utilize the same core measures selected from the baseline survey to ensure consistency across data collection visits. The app allows participants to report their HIV self-testing results and upload a photo for validation of results. Figure 3 displays the study app home screen, participant study timeline, and access to the survey and entry page for the home HIV self-test results.

Participants who experience HIV seroconversion during the study are asked to complete a facility-based visit for confirmation of HIV status (if HIV infection was identified during a Web-based visit) and referral. These participants are offered an additional follow-up visit 3 months after identification of seroconversion to assess uptake of referrals and engagement in the HIVCC (Figure 1). Participants are asked to complete a brief survey to answer questions about recent engagement in HIV care. This also serves as an opportunity for the study to provide additional referrals for any participant who has not yet accessed HIV care.

### Measures

Baseline (Aims 1 and 2) and longitudinal surveys (Aims 3 and 4) are informed by the situated vulnerabilities framework [1]. Validated measures for use among TW or transgender populations have been incorporated into the survey [77], and wherever possible, we have selected measures previously tested with diverse populations to minimize cultural bias and to maximize comparability to other studies.

#### Survey Measures

All surveys, including facility-based and Web-based, are self-administered. We adapted the Rapid Estimate of Adult Literacy in Medicine – Revised, a brief literacy screening instrument used to assess an adult’s ability to read common medical words, to include terms relevant to TW that are included in the survey (eg, pill, hormones, augmentation, and hepatitis) [78]. At the baseline visit, participants are asked to read 10 terms aloud; those who correctly read 7 or fewer are asked to participate in an interviewer-administered survey. Staff are also available to offer interviewer-administered surveys to participants with other needs that limit the participants’ ability to self-administer (ie, visual impairment or discomfort with using a handheld tablet device).

Individual-level measures collect self-reported data on demographics, including zip code of residence, age, race, ethnicity, education, employment status, health insurance, housing, mobility, and history of detention/incarceration; gender affirmation surgery, care, and exogenous hormone use, including age of initiation, based on the US TransPop Survey [77]; sexual health history and access/uptake of HIV services (including postexposure prophylaxis; PEP and PrEP), based on the Centers for Disease Control and Prevention’s (CDC’s) National HIV Behavioral Surveillance [79]; and primary care, mental/ behavioral health, social services utilization, and barriers and facilitators to care [14,15,80]. Substance use is measured by the Alcohol Use Identification Test Consumption Questions and the Drug Abuse Screening Test with additional measures for injecting drug use [81-83]; mental health symptoms including psychological distress (Kessler-6), posttraumatic stress disorder (PC-PTSD), and history of suicidal ideation and attempt [84]; and gender pride, affirmation, and dysphoria and transgender adaptation and integration assessing adjustment experiences specific to gender identity[85]. To assess Aim 4, which focuses on engagement of HIV-uninfected participants in the HIVPC, measures of linkage of HIV-uninfected participants to prevention services, types of services, retention in such services, and adherence to prevention interventions are also included [86].

Interpersonal measures include sexual relationships and behaviors measured via an adapted version of the AIDS-Risk Behavior Assessment [87,88] to assess self-reported, detailed partner-by-partner sexual behavior information by partner type and HIV status. This measure was updated for the potential incorporation of study data into mathematical modeling. Substance use in the context of HIV acquisition risk and “triggers” for sexual risk taking, which have been adapted for transgender populations to ensure gender-affirmative assessment of sexual risk, are included [89,90]. Measures of intimate partner violence (IPV) and nonpartner violence (NPV ) victimization, respectively), including physical, sexual, and psychological violence and control, are based on an adaptation of the Conflicts Tactics Scale [91].

Structural-level measures include social marginalization and stigma, focusing on experiences of enacted and anticipated discrimination, were adapted from the Intersectional Discrimination Scale [92]. Social capital, with focus on participation in the local community; social agency; feelings of trust and safety; neighborhood connections; and friends/family connections are included from subscales from the Social Capital Scale [93,94]. Interactions with the justice system and measures of immigration status, citizenship, and interactions with immigration detention are included. Finally, upon further recommendation by the CAB, an additional measure of food insecurity, adapted from United States Department of Agriculture Food Insecurity measures, is now included in the survey [95]. Surveys for participants with known HIV infection or who subsequently seroconvert include questions about engagement and retention in HIV care; initiation of antiretroviral therapy and adherence; CD4 and viral load testing; self-reported viral suppression, as defined by the National HIV/AIDS Strategy; and access to additional services for HIV infection [96-98]. Participants are also asked to provide consent to a medical record review for an extraction of their most recent confirmatory test, CD4, and viral load results.

#### Biological Measures

All HIV rapid testing is conducted following completion of the survey. HIV self-testing is performed by baseline participants including, for confirmation purposes, those who report known HIV infection. Participants self-administer the OraQuick self-test (OraSure Technologies) in the study facility during the baseline visit with support from trained study staff. This provides an opportunity for staff to train participants and answer any questions on the use of HIV self-test kits. HIV test results are available within 20 min. All participants with a positive HIV self-test undergo confirmatory testing according to CDC recommendations [99], and the patients are referred to a local and affirming HIV care facility of their preference.

**Figure 4:**
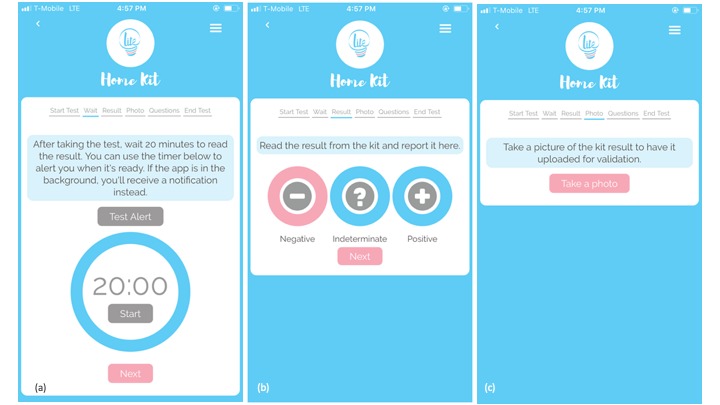
HIV testing component of the study app: (a) HIV test timer, (b) HIV test results entry, and (c) photo upload for HIV test validation.

The HIV self-test is used for every 3-month visit (facility-based or Web-based) by cohort participants to support measurement of the primary outcome of interest (seroconversion). Participants completing Web-based study visits are asked to use the study app (Figure 4) or a secure URL to implement self-testing and report their HIV test results. The study app provides a video link to provide additional instructions to participants and a timer to guide implementation and prompt users to record the test result after 20 min. Participants are asked to interpret the results of the test and upload a photo of the test. Any participant with an unclear or otherwise invalid photo is asked to attempt to upload the photo again or repeat the HIV test. Auto notifications are sent immediately to the study staff for cohort participants who report a positive or indeterminate HIV self-test conducted at home; these participants are requested to come immediately to the study facility for confirmation and referral. This component of the app also includes measures of HIV self-testing acceptability for all participants who perform the HIV self-test on their own.

The oral point-of-care HIV test, OraQuick, was approved for self-testing use in the United States by the US Food and Drug Administration in 2012 [100]. OraQuick instructions are available in both English and Spanish. Test performance by untrained users is estimated to have a sensitivity of 99.9% and a specificity of 91.67% [101]. Participants are provided with the 24-hour OraQuick Support telephone number as well as the study coordinator (SC) number if participants have questions or have difficulty administering the test or interpreting the result. In addition to reducing staff time and involvement in HIV testing, self-testing has the promise of allowing TW to take greater control over their own health by putting the choice of when and where to test in their hands [102]. Study staff who can provide support may address the limitations of over-the-counter self-tests and improve testing behaviors. To our knowledge, there are no published data available on use of HIV self-testing or impacts of self-testing on undiagnosed infections among TW in the United States, although pilot studies have suggested high acceptability among TW [103].

Blood samples are collected at baseline and at 12 and 24 months. Plasma are collected and shipped to JHU to identify individuals with recent infection and estimate incidence at baseline by using a multiassay algorithm [104]. HIV incidence estimates can be greater than twofold lower in a longitudinal cohort than the underlying population [105,106] because of behavioral modification for those who enroll (Hawthorne effect) [58] and differential loss to follow-up by risk. We will also compare the individuals who appeared as recently infected at enrollment [107] with those who seroconverted during follow-up to determine if behavioral modification or differential loss to follow-up resulted in the difference in incidence [108]. In addition, the biospecimens at baseline and those collected during follow-up will be stored for future phylogenetic analysis, until additional funding become available.

Self-collected specimens to test for STIs are collected from all participants at baseline and at 12- and 24-month visits, including among HIV-infected baseline and HIV-uninfected cohort participants. Urine samples as well as swabs collected at anorectal and vaginal (for those with vaginoplasty) sites will be self-collected to measure presence of gonorrhea and chlamydia infection. Serum treponemal syphilis testing and rapid plasma reagin (RPR) testing with quantitative RPR titers are used to test for syphilis infection, per local protocols. STI testing is conducted locally at facility-based visits. These STI tests serve as a biologic proxy measure for HIV risk.

#### Recruitment Process Measures

Aim 1 focuses on the efficiencies of recruitment methods. Data on the number of banner ads and postings, impressions (number of online views), and click throughs (number of times the ad was clicked) and data on the number of visits to the study website will be collected for all technology-infused recruitment. For recruitment from gender-affirming events, the number of events, number of participants at each event, and number of candidates accessing study information will be collected. For peer referral, information on the number of unique coupons provided to each participant, number of declined coupons, and number of unique coupons returned to the study staff will be collected. For clinic-based referral, the number of unique TW clients utilizing the clinic during the recruitment period and number of referrals or fliers distributed will be collected. For all recruitment methods, time from recruitment initiation to enrollment, costs associated with recruitment, the number of scheduled enrollment appointments, and the numbers of eligible participants (by HIV status) will be collected by recruitment method. Baseline survey participants will be asked to report how they learned about the study.

### Quality Assurance/Quality Control Plan for All Study Phases

Before study implementation, a study manual of procedures was developed, which included the full study protocol as well as study background, design, organizations, recruitment techniques, informed consent, clinical procedures, participant safety, instructions for entering data and using tablets, follow-up procedures, and study definitions. The manual of procedures was then adapted to each site for additional details related to unique aspects of the facility where research is conducted. Updates and clarifications are made as necessary over the course of the project, with revisions available to all staff. All staff are required to undergo training on the overall research objectives and protocol, transgender health, and HIV, along with human subjects training. Clinical staff are required to complete additional training on clinical procedures, confidentiality and privacy, and Good Clinical Practice. Refresher training is scheduled on an annual basis. Weekly calls are held with SCs at each site to discuss adherence to the study protocol, enrollment progress, and experiences with retention.

To ensure consistency in the survey data collection and minimize social desirability bias, we use a computer-assisted self-interview methodology, which includes self-administered tablet-based surveys during facility-based visits and self-administered app-based or Web-based surveys during Web-based study visits. The use of the self-administered tablet-based survey at the facility-based baseline visit allows participants to become accustomed to the Web-based survey and ask for assistance from the study staff. The survey instruments were pilot tested before implementation, and logic checks and skip patterns are built into the program to minimize errors in data capture. Security features of the Web-based and app-based survey allow for data to be saved regularly as participants advance through the survey questions, preventing unintentional loss of data should there be a loss of internet connectivity. Data are reviewed weekly to check for consistency in the data capture processes and assess missingness of data.

### Participant Tracking

**Figure 5:**
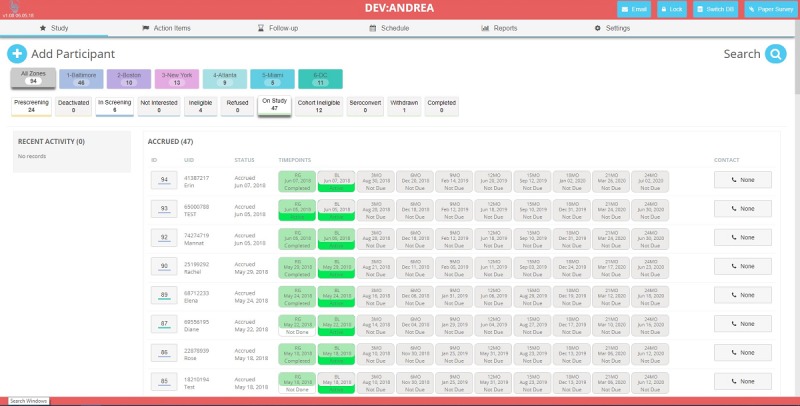
Participant database (note: image is a development interface and displays test subjects, not actual participant data).

Cross-sectional and cohort participants are tracked using a Web-based, Health Insurance Portability and Accountability Act–compliant system hosted by JHU central information technology which allows for facility-based data collection and participant tracking over the course of the study (Figure 5). This system has been used in other prospective studies by the JHU team [109].

The Web-based tracking system is customized and configured by the study team to fit the study protocol. Each site has role-based and site-based access to their data, with JHU having full administrative access to all data. It has also been expanded to provide the additional cohort participant app, with an optional URL platform for nonsmartphone users. At enrollment, a unique identifier is generated for participants to log in and access the app and associated surveys. To assist with the recall of the identifier, cohort participants are provided with a study gift (eg, cellphone cardholder) with a study card that includes their study identifier. SCs assist cohort participants with download and installation of the app during enrollment. Participants who encounter problems with subsequent installation (if phone is replaced) or log-in are encouraged to call the study telephone number for support. The participant app is locked on the device once installed, and it requires the entry of the assigned study ID for it to be accessed. The app is Global Positioning System (GPS) enabled, although participants may opt out, to allow for measurement of participant mobility over the duration of the study. The tracking system issues automated push notifications and SMS text messages to the participants’ device for follow-up visit reminders.

All data collected by the tracking system and participant app are encrypted in transit from the device to the server. All the identifying information collected from participants is stored encrypted within the database using the AES 256 bit protocol. Only the research staff requiring access to identifying information for retention purposes has access to protected health information through the tracking system.

### Study Retention

Meticulous tracking and retention procedures increase the likelihood that attrition is random and not systematic during study implementation. We use tracking and retention procedures that were proven effective in previous studies. The SC at each site devotes at least 50% time to recruiting and tracking subjects. The SC and other research staff collect and regularly update extensive email/phone/social media contacts, as well as the addresses and contact information of the 3 people that participants believe could help us locate them and will telephone/email/text participants to remind them of their appointment. Automated study visit reminders are also built into the participant tracking system and study app.

Culturally appropriate stepped financial incentives are also provided at each follow-up to encourage continued participation. Depending on the site, additional nonmonetary strategies to improve retention include the following: (1) an anonymous raffle for study participation, (2) provision of links to other non-HIV gender-affirming care and services available during facility-based visits and on the study website and app, (3) a Facebook page on which participants can communicate with each other and staff and post events, and (4) retention events focused on providing non-HIV services of interest (eg, legal name and gender-marker change workshops for identification documents, spa days, and other social events). Retention events are typically held within the community to keep participants engaged in the project.

Facilities provide a range of times of day and days of the week for participants to come to their assessment visits, including evening hours and weekends to support participation by individuals who work or are in school full time. Clinical partners also have excellent reputations within their communities, and they are known for being nonstigmatizing and ensuring privacy and confidentiality, which assists with retention. We also have an extensive network of transgender-focused organizations and clinical care providers in each of our partnering metropolitan areas who serve as referral centers for health needs. Finally, we endeavor to hire study staff who are from the TW or lesbian, gay, bisexual, and transgender community and who have nursing or public health experience, to ensure cultural competence and optimize acceptability of the study.

Study retention is operationally defined as not missing more than 3 consecutive study visits. After 2 missed visits, SCs initiate the retention protocol to track participants through individual and friend/family contacts and calls to local facilities to identify potential incarceration. Participants who express a desire to prematurely exit the study are asked to complete a participant withdrawal questionnaire (on the Web, by phone, or in person) to assess basic characteristics at study exit (eg, HIV status and PrEP use), reason for withdrawal, and whether additional referrals are needed. The study team assesses whether the reason for withdrawal can be addressed, and then the study team will offer resolution, within the limits of the protocol, to the participant. Cohort participants who are lost to follow-up or who have previously withdrawn and request to reenter the study are screened and reassessed for eligibility, and if the timeline allows, they are administratively considered for reenrollment.

### Statistical Considerations

#### Sample Size

We anticipate that 1750 individuals will be screened (approximately 350 per city) from years 1 to 2. Assuming 10% of those screened are ineligible and have an HIV prevalence of 30%, TW enrolled at the end of Year 2 will include 1100 HIV-uninfected TW (approximately 220 per city) and 475 HIV-infected TW in the baseline, cross-sectional comparison group.

**Figure 6:**
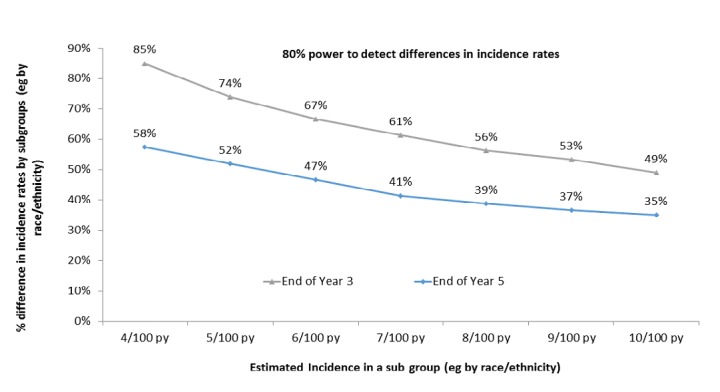
Power to detect public health–relevant differences in HIV incidence by subgroups. py: person years.

We conservatively estimated that 700 pys of follow-up accumulated from Years 1 to 2 during enrollment. Incorporating a loss of 15% of follow-up time per year (from a combination of loss to follow-up and mortality), we estimate that we will accumulate 935 pys, 795 pys, and 676 pys in Years 3, 4, and 5, respectively (>3000 pys total). To compare the incidence of 2 subgroups (eg, incidence by black vs nonblack race), assuming 2-sided test statistics and 5% Type 1 error 80% power, we have sufficient power (80%) to detect public health–relevant differences in HIV incidence by various participant characteristics (Figure 6).

##### Sample Size Considerations

HIV incidence in TW is the primary study outcome of interest. With respect to the baseline, cross-sectional incidence estimate, a sample of 1575 individuals at baseline with 30% prevalence and estimated cross-sectional 8% incidence would identify 37 “recently” infected individuals with a 95% CI of 4.7-11.5 using the validated algorithm [104].

Study attrition is tracked using data visualization techniques, stratifying important metrics over time (such as the number enrolled and proportion lost to follow-up) by site. Monthly updates to these figures will help to detect problems and initiate a timely response to ensure at least 1000 TW are enrolled in the cohort and to reduce loss to follow-up. Statistical analyses of attrition will be conducted, including a comparison of demographic characteristics, social factors, and HIV risk behaviors among those retained versus lost to follow-up. Survival analyses will be used to investigate factors associated with loss to follow-up.

#### Analytic Plan

Data management and statistical analysis are led by statisticians and data managers in the John Hopkins Bloomberg School of Public Health (JHSPH) Statistics in Epidemiology (STATEPI) group (which serves the North American AIDS Cohort Collaboration on Research and Design and the Multicenter AIDS Cohort Study / Women’s Interagency HIV Study Combined Cohort Study, which are longitudinal cohorts of adults with, and at-risk for, HIV infection) with oversight by study investigators (ALW, SR, and KNA). Analyses essential to the proposed aims include tracking and investigations into attrition, comparison of HIV-infected and HIV-uninfected TW at baseline, both HIV incidence and time-to-event analyses to better understand drivers of HIV transmission, and serial cross-sectional looks at the HIVPC and HIVCC over calendar time.

Statistical analysis throughout the study addresses the overarching specific aims and hypotheses. Aim 1 analyses focus on exploring optimal recruitment methods and the efficiency of each method. Data visualization techniques are being utilized to compare efficiency of recruitment methods in terms of process measures of time, cost, and response rates. Additional descriptive analyses compare quantitative measures of acceptability and will also assess diversity and potential biases of the samples recruited, in terms of demographics, risk behaviors, and HIV status. Differences are not statistically compared across recruitment methods, given heterogeneity of recruitment methods and process measures.

Aim 2 analyses include descriptive statistics to compare demographic and behavioral risk profiles among HIV-uninfected and HIV-infected baseline participants as well as to assess baseline HIVPC and HIVCC among HIV-uninfected and HIV-infected participants, respectively. Log binomial models (or, when such models fail to converge, we will approximate with Poisson regression models with robust variance) are used to estimate HIV prevalence ratios and 95% CIs for demographic and risk characteristics; subgroup analyses will be conducted, stratified by site. Latent class analysis will be further used to explore profiles of HIV-infected and HIV-uninfected participants at baseline [110]. Statistical analysis is completed using Stata version 14 (StataCorp) [111], Mplus (Muthen & Muthen) for latent class analysis, and RDS Analyst in R software (University of California, Los Angeles) for visualization of peer-recruitment networks (including homophily and depth).

Aim 3 analyses will focus on differences in HIV incidence during follow-up (and 95% CIs) among the HIV-uninfected cohort will be estimated using Poisson regression models to investigate important predictors, including (but not limited to) age, race/ethnicity, and geographic location. Observed HIV incidence from the cohort will be compared with baseline cross-sectional incidence to assess for differences in incidence and potential mechanism for such differences (eg, differences in participant profiles and behavioral modification). Time-to-event survival models will be used to estimate the risk of HIV seroconversion for the predictors. A scan statistic will be used with the zip code of residence and GPS data to detect seroconversion event clusters in time or space (or both) for identification of potential microepidemics. The scan statistic is commonly used to identify the most likely cluster location, where a significant *P* value (*P*<.05) indicates that it can be concluded that the most likely location of clustering was unlikely under test assumptions [112]. Statistical analyses will be performed using Stata 14 (StataCorp) and SatScan (Kulldorff).

Analyses for Aim 4 will focus on the steps in the HIVPC and HIVCC are investigated among HIV-uninfected and HIV-infected TW, respectively, using a serial cross-sectional approach to determine trends in these important frameworks. To make these estimates most useful for policy and program, the steps are estimated among those under observation in specific calendar years (ie, in 2019, 2020, and 2021).

For analytic refinements, we will make use of modern missing data techniques [113], with particular emphasis on multiple imputation methods, which provide a useful umbrella for handling different forms of missing data including coarsely measured [114] or mismeasured [115] variables. Adaptation of these methods to survival analyses will be similar to techniques implemented in R/MICE and SAS/IVEware [116-118]. These methods are appropriate when data are not missing at random and missing data occur in multiple variables simultaneously. For evaluating the impact of unmeasured confounders, we will make use of sensitivity analyses techniques [119] previously used to determine the impact of unmeasured confounders by the strength of the confounder.

### Participant Protections

Scientific measurement of HIV infection, as well as gender identity, sexual identity and behavior, and stigma, is inherently sensitive in nature. All study activities are developed with attention to protection of participant privacy and confidentiality and in discussions with the study CAB. All participant study data are retained separately from participant identifiers; only study staff responsible for scheduling or referrals have permissions to access participant identifiers. The electronic data system has been developed with careful attention to security, including password protection and no obvious information that the app is developed for TW or for HIV research. Participants are provided with clear instructions on how to use, discretely store, and report results from the HIV self-test as well as how to contact the study staff in the event of a positive test result. Given concerns of stigma and discrimination, only referrals to clinics or community based organizations that have been vetted and determined to be gender affirming are provided. Finally, the CAB has been consulted throughout all phases of the study to ensure acceptability of methods among the TW community. Study activities follow a single centralized Institutional Review Board (IRB) procedure and have undergone review and approval by the Johns Hopkins School of Medicine IRB. All collaborating organizations and institutions rely on this approval.

### Scientific Rigor and Reproducibility

Rigor and reproducibility are ensured throughout all phases of the study by the stringent application of the scientific method to minimize bias in design, methodology, analysis, and interpretation of results, and they will follow methods successfully used by *MACS, WIHS*, and *LifeSkills* cohorts [88,120,121]. These methods include but are not limited to the following: inclusion of a baseline, cross-sectional sample of HIV-infected TW against which the risk profile of the HIV-uninfected cohort may be compared; sample size estimation based on power to detect differences in incidence across subgroups and to detect differences estimated during cross-sectional incidence testing and longitudinal measures of incidence; attention and efforts to maximize study retention; attrition analysis as well as assessment and methods to address missing data; use of statistical measures that have demonstrated applicability in HIV incidence estimation and descriptive analysis; and documented quality assurance/quality control by the data collection/management group; as well as standardized methods utilized by the JHSPH *STATEPI* group. Several steps are undertaken to ensure reproducibility of the study results, including publication of the study protocol and methodology and data archiving of deidentified data for public use; a scientific steering committee, composed of the multiple PIs, the PIs of the site cohorts, and the primary data coinvestigators; a concept sheet process to allow for input from all and to create opportunities for research by all investigators, including external investigators and junior faculty members; development of analytic summary files to ensure key variables are put into a scientifically appropriate form and used consistently in analyses; and development of analytic standards for analyses, including adjustment for differences by cohort, sensitivity analyses stratified by study site.

## Results

Formative research was conducted between August 2017 and January 2018, and findings have been described elsewhere [59,66]. These included 5 Web-based synchronous computer-mediated focus groups conducted in English (n=33) and 2 Web-based synchronous computer-mediated focus groups that were conducted in Spanish (n=8). The geographically diverse sample, which spanned all 6 cities, had a mean age of 41.1 (SD 13.6) years; 66% (27/41) of the participants identified as people of color, and 29% (12/41) of the participants identified as Hispanic/Latina. Participants provided input into the study name and branding, recruitment materials and messaging, and considerations for implementation of HIV self-testing. Participants also described past experiences of HIV research participation, which largely described the social and economic factors that shaped their experiences. Barriers to and negative experiences in HIV research participation included limited research opportunities, mistrust, fear of mistreatment, concerns about safety and confidentiality, competing priorities, and HIV stigma. Facilitators to and positive experiences with HIV research participation included peer involvement and engagement, monetary and nonmonetary incentives, flexibility and choices, multiple modalities and methods, and transcenteredness [66].

In March 2018, the cohort was launched at the Baltimore and Boston sites, followed by launches of the cohort in the remaining cities in April and May 2018. This staggered approach was established to allow for any troubleshooting of study implementation procedures, the CTMS, and study app before the full launch in other sites as well as for final IRB reliance agreements to be established. As of March 20, 2019, 795 TW were screened and 739 TW were enrolled in and completed the baseline study visit (median age 32 years, interquartile range; IQR 25-42 years). Majority of the participants are racial/ethnic minorities, with 45.1% (333/739) of the participants identifying as black and/or 27.8% (206/739) identifying as Hispanic/Latinx.

Of these baseline participants, 206 (27.8%, 206/739) were found to be living with HIV at baseline and 480 (64.9%) were HIV-uninfected and met behavioral risk criteria and were enrolled in the cohort (cohort median age: 29 years, IQR 24-37 years). This contributed to a total 245.4 pys in the cohort as of March 2019. A total of 17% (126/739) of the baseline participants were identified with lower levels of literacy, requiring interviewer-administered surveys and HIV tests, as well as in-person visits. Other participants have requested in-person visits, reportedly for the purposes of having a safe and private place to participate in study activities, comfort in having a staff member perform or be present for HIV testing, or because the participant wanted to see or spend time with study staff.

Baseline participants have entered the study through a variety of recruitment methods, including peer referral (38.9%, 288/739), referral from a health facility (40.0%, 296/739), referral from a community-based organization (12.0%, 89/739), study flier (10.9%, 81/739), Facebook (7.9%, 59/739), dating apps (4.0%, 30/739), or other websites (4.0%, 30/739), such as Craigslist, Backpage, and Eros. However, study recruitment has progressed overall at a rate lower than expected and with lower proportions recruited online than anticipated. The use of advertising campaigns in dating apps to recruit participants appeared to reach a large number of individuals (1,390,827 impressions and 11,629 clicks on the study website during geotargeted campaigns via 1 dating app), but it resulted in only 24 enrollments to date. This is likely attributable to the fact that TW may use dating apps that are developed for heterosexual populations, as well as people in same sex relationships, with no single app being uniquely for or in high use by TW, thus limiting the number of those who can be reached through advertisements via these apps. Furthermore, challenges in communication with dating app representatives, low transparency and barriers to recruitment data, and cost associated with recruitment via dating apps suggest that these are not efficient methods for recruitment of TW for research. Given these challenges, we have broadened recruitment to advertising campaigns via Facebook, Reddit, and Google Ads, as well as sharing information about the study through media interviews [122-125].

## Discussion

This study is responsive to increasing research interest in technology-enhanced methods for cohort research, particularly for hard-to-reach populations. The cohort utilizes innovative methods that harness technology alongside traditional methods and facility-based visits to develop the first multisite US cohort to assess HIV risk among one of the most affected populations. Study findings have important implications for informing future HIV prevention and care research, including identification of optimal recruitment and retention methods, estimating HIV incidence and identifying risk factors for HIV acquisition, and ultimately informing the development of acceptable HIV prevention and care efforts for TW in the United States. Importantly, the diversity of literacy, technology use, and overall socioeconomic situations in this sample of TW highlights the need to balance study rigor with use of flexible methods to ensure that with increasing technology, those living in the most vulnerable contexts are not excluded from research.

Limitations of the study must be recognized. First, the sample size is relatively small compared with other national cohorts that comprise larger, less stigmatized populations. However, the use of multiple partnering sites with strong ties to the TW community, partnership with a CAB, and use of mixed-format recruitment and data collection methods are anticipated to encourage recruitment and retention across sites. As additional funding is identified, new sites and organizational partners may also be added. Although the pooled contribution of cohorts from multiple sites adds strength to the study, pooled results can provide misleading or overly smoothed inferences. Our analytic “best practices” protocol includes examining data for each cohort to understand the magnitude of heterogeneity in estimates. Statistical methods for determining cohort interactions and combining pooled results [113] will be utilized. Furthermore, we will continue to use sensitivity analyses to examine the influence of individual cohorts on specific results, such as omitting cohorts systematically by use of jackknife methods with unequal partitions [114]. Finally, the use of cross-sectional incidence testing has been incorporated into the study to support and assess longitudinal incidence estimation as well as to assess for any Hawthorne effect associated with participation in the cohort.

This study establishes a multisite, longitudinal cohort of TW in eastern and southern United States, targeting 6 high-risk metropolitan areas: Baltimore; Washington, DC; Boston; New York City; Atlanta; and Miami, and it is now one of the largest cohorts specifically comprising TW in the United States. The goal is to characterize risk factors for HIV infection, access to biobehavioral HIV prevention methods, and linkage to care for those who HIV seroconvert for the purposes of informing evidence-based and acceptable interventions to reduce HIV incidence for this at-risk population. The cohort will include a racially, ethnically, and culturally diverse sample of TW, supported by technology-based recruitment and retention methods. The study is designed to (1) answer methodologic questions about appropriate and effective recruitment and retention methods for TW, as well as potential biases in these methods; (2) provide HIV incidence estimates and, through the use of both cross-sectional and longitudinal incidence measures, assess potential influences on incidence estimations; (3) identify individual social and structural risk factors for HIV acquisition; and (4) evaluate engagement in the HIVPC and HIVCC among HIV-uninfected and HIV-infected TW. We anticipate that the cohort study will serve as a platform upon which pilot interventions, studies of sexual partners, cohorts of TW youth, additional geospatial and phylogenetic analysis to further contextualize hot spots, mathematical modeling to assess the impact of promising interventions, and analysis of the role of exogenous hormone use in HIV infection can be built. Collectively, these findings will inform optimal components of promising interventions to reduce HIV incidence among TW.

## Appendix

Multimedia Appendix 1 Peer-reviewer report from the National Institutes of Health.

## Abbreviations

BCH: Boston Children’s Hospital
CAB: Community Advisory Board
CDC: Centers for Disease Control and Prevention
CFAR: Centers for AIDS Research
CMC: computer-mediated communication
GPS: Global Positioning System
GSN: geosocial networking
HAHSTA: HIV/AIDS, Hepatitis, STD, and TB Administration
HIVCC: HIV Care Continuum
HIVPC: HIV Prevention Continuum
IQR: interquartile range
IRB: Institutional Review Board
JHSPH: John Hopkins Bloomberg School of Public Health
JHU: Johns Hopkins University
LITE: Leading Innovation in Transgender Health and Empowerment
PI: principal investigator
PrEP: pre-exposure prophylaxis
pys: person years
RDS: respondent-driven sampling
RPR: rapid plasma reagin
SC: study coordinator
SMS: short message service
STATEPI: Statistics in Epidemiology
STI: sexually transmitted infection
TW: transgender women
