# Supplementary material for: American Cohort to Study HIV Acquisition Among Transgender Women in High-Risk Areas (The LITE Study): Protocol for a Multisite Prospective Cohort Study in the Eastern and Southern United States
Source: JMIR Res Protoc. 2019 Oct 3;8(10):e14704. doi: 10.2196/14704 (PMC6802485; doi:10.2196/14704)
Supplement: Multimedia Appendix 1 [file resprot_v8i9e14704_app1.pdf]

SUMMARY STATEMENT

(Privileged Communication)

PROGRAM CONTACT:
Lori Zimand
240-627-3212
lzimand@niaid.nih.gov

Release Date: 05/08/2017
Revised Date:

Application Number: 1 UG3 AI133669-01

Principal Investigators (Listed Alphabetically):

REISNER, SARI
WIRTZ, ANDREA (Contact)

Applicant Organization: JOHNS HOPKINS UNIVERSITY

Review Group: ZAI1 AL-A (M1)
National Institute of Allergy and Infectious Diseases Special Emphasis Panel
Limited Interaction Targeted Epidemiology (LITE) to Advance HIV Prevention (UG3/UH3)
AIDS - EXP. REV.

Meeting Date: 04/19/2017
Council: MAY 2017
Requested Start: 07/01/2017

RFA/PA: AI16-031
PCC: A27I
Dual PCC: 9A-ASG
Dual IC(s): MH, HD

Project Title: American Cohort to Study HIV Acquisition among Transgender Women in High Risk Areas
SRG Action: Impact Score:27
Next Steps: Visit https://grants.nih.gov/grants/next_steps.htm
Human Subjects: 30-Human subjects involved - Certified, no SRG concerns
Animal Subjects: 10-No live vertebrate animals involved for competing appl.
Gender: 2A-Only women, scientifically acceptable
Minority: 1A-Minorities and non-minorities, scientifically acceptable
Children: 3A-No children included, scientifically acceptable

Project Year	Direct Costs Requested	Estimated Total Cost
1	1,146,568	1,728,996
2	1,192,758	1,798,650
3	1,130,062	1,704,106
4	1,116,383	1,683,478
5	1,080,787	1,629,800
TOTAL	5,666,558	8,545,030

ADMINISTRATIVE BUDGET NOTE: The budget shown is the requested budget and has not been adjusted to reflect any recommendations made by reviewers. If an award is planned, the costs will be calculated by Institute grants management staff based on the recommendations outlined below in the COMMITTEE BUDGET RECOMMENDATIONS section.

1UG3AI133669-01 Wirtz, A

DATA SHARING PLANS
SCIENTIFIC REVIEW OFFICER'S ADMINISTRATIVE NOTES

RESUME AND SUMMARY OF DISCUSSION: This outstanding competitive application entitled “American cohort to study HIV acquisition among transgender women in high risk areas” is submitted in response to RFA-AI-16-031: Limited Interaction Targeted Epidemiology (LITE) to Advance HIV Prevention (UG3/UH3) by the Johns Hopkins University with Dr. Andrea Wirtz as the Program Director (PD). The application proposes to establish a multi-site longitudinal study of transgender women (TW) in the Eastern and Southern part of the US to assess the risk factors this high risk subpopulation cohort is exposed to. The cohort will include racial and ethnically diverse samples of TW. The multi-site will include Boston, New York City, Baltimore-Washington area, Atlanta and Miami. Two aims are proposed in the UG3 and UH3 phases of the application. In the UG3 phase, technology-based recruitment methods will be used to enroll HIV-, high risk TW in the five metropolitan areas. Efficiency of the recruitment tools also will be evaluated. Data on the recruited individuals such as their social, network, demographic, behavioral patterns and lifestyles will be collected. In the subsequent aims in the UH3 phase, HIV seroconversion incidences, risk factors associated with these cases as well as information about compliance rates with care acceptance, adherence and treatment discontinuation of newly HIV infected patients will be collected.

One strength of this application is the reviewers’ consensus recognition that TW is a relevant cohort to study HIV infection. Additionally, the research team led by Dr. Andrea Wirtz is an experienced group with extensive working knowledge about TW’s risk factors. A proposed list of measurements for analysis from the collected data is comprehensive and includes social, network, demographic, behavioral patterns and lifestyles. The application also proposes to use various recruitment methods. This plan is a strength because they will potentially maximize the recruitment numbers of this population. Furthermore, some of the methodologies are untested on TW and thus, a multi-prong approach is necessary for recruitment in case one or two methods fail to recruit the desired number of participants. Technology-driven recruitment approaches will be used and the research team will investigate if such approaches will be more effective than the traditional non-technological methods. The application also proposes an innovative method to identify recruits such as fingerprinting the submitted data by the participants, although no alternative plans are included in case of failure.

Weaknesses of the application include the lack of a detail description about some of the measurements that the research team plans to use. Specifically, there is a concern that some measurements may require adjustment because they have not been used on this cohort before. It is the consensus of the reviewers that the application does not reflect an awareness that the proposed methodologies may need to be modified to reflect diverse cultural differences; the type of language that the questionnaire uses may need to be modified depending on the regions within the US targeted for TW recruitment. Although the list of measurements is comprehensive, the sheer number of the measurements is enormous. Concern is raised if it is feasible and if it will affect participant retention. The proposed Community of Advisory Board (CAB) appears to be underutilized because its function is not well described in the application. There also is concern about the frequency of data collection from the participants every 3 months - is 3 months too often a contact between the research team and the individual? The modest budget proposed for the UG3 phase is questionable for feasibility. Finally, there is doubt that targeting TW and subsequently treating this group, even if their projected recruitment numbers are reached, will be impactful to the HIV epidemic.

Based upon the evaluation of the scientific and technical merits, the application received an Overall Impact score of 27.

DESCRIPTION (provided by applicant): In the United States (U.S.), transgender women (TW) are one of the populations most affected by HIV infection. The high prevalence of HIV infection among U.S. TW is driven by, and/or concomitant with, structural barriers that limit access to HIV prevention, care, and health services. Despite an emergence of research to characterize the HIV epidemic among TW, the majority of studies are cross-sectional designs and typically include small sample sizes, often subsuming TW among broader risk groups, such as men who have sex with men. These practices have thwarted identification of acceptable, effective recruitment and study methods for use among TW and prevented temporal assessment, causal inference, and generalizability of study findings to the TW population. To date, there is no robust estimate of HIV incidence and no intervention with evidence of efficacy for the prevention of HIV acquisition among TW. The proposed American Cohort study will address these limitations by establishing a multi-site, longitudinal cohort of TW spanning eastern and southern U.S. (Boston, New York City, Baltimore-Washington, Atlanta, and Miami metropolitan areas) to characterize HIV incidence and risk factors for HIV acquisition, access to biobehavioral HIV prevention methods, and linkage to care for those who HIV seroconvert. The cohort will include a racially/ethnically and culturally diverse sample of TW, supported by the use of technology-infused recruitment and retention methods. The specific aims are: 1) To determine the efficiency and acceptability of novel, technology-infused recruitment methods to enroll HIV- uninfected TW into a prospective cohort. 2) To describe the demographic, socioeconomic, behavioral, and physical and mental health profiles of HIV-uninfected TW in the first, multi-site cohort of TW in the eastern and southern U.S. 3) To estimate HIV incidence among TW in high-risk eastern and southern U.S. areas, trends in incidence, and associated individual, social, and structural risk factors. 4) To estimate the HIV Prevention Continuum among HIV-uninfected participants, and the HIV Care Continuum among newly HIV-infected TW. To achieve these aims we will recruit 1,750 TW who will be enrolled in either a HIV-uninfected cohort or an HIV-infected cross-sectional comparison group. These data will be used to assess differences between cohort participants and the wider population and for cross-sectional incidence estimation at enrollment. The HIV- uninfected cohort will be followed for at least 24 months to estimate HIV incidence, trends, and risk factors for HIV acquisition. Cohort participants who seroconvert will be followed for an additional 6 months to assess prospective engagement in the HIV Care Continuum. Study findings will provide critical epidemiologic parameters for future HIV prevention research among TW, provide a platform upon which other research questions can be explored, and inform the development of evidence-based and acceptable HIV interventions to reduce HIV acquisition among TW in the U.S.

PUBLIC HEALTH RELEVANCE: The American Cohort study proposes to establish multi-site, longitudinal cohort of transgender women in the eastern and southern U.S. (Boston, New York City, Baltimore-Washington, Atlanta, and Miami metropolitan areas) to characterize risk factors for HIV acquisition, access to biobehavioral HIV prevention methods, and linkage to care for those who HIV seroconvert. The cohort will include a racially/ethnically and culturally diverse sample of TW, supported by the use of technology-infused recruitment and retention methods. Study findings will provide vital epidemiologic parameters for future HIV prevention and care research among transgender women, offer a platform upon which other research questions can be explored, and inform the development and testing of evidence-based and acceptable HIV prevention interventions for this population.

CRITIQUES: The written critiques of individual reviewers are provided in essentially unedited form below. These critiques were prepared prior to the meeting and may not have been revised afterwards. The "Resume and Summary of Discussion" above summarizes the final opinions of the committee.

CRITIQUE 1

Significance:	3
Investigator(s):	2
Innovation:	4
Approach:	4
Environment:	1

Overall Impact: The proposed project has the potential to contextualize the lives of an understudied and highly vulnerable population. If successful in reaching the targeted recruitment in the first phase, the methods set out for the second phase are appropriate. Thus, the likelihood exists for a sustained influence in the form of future intervention tailoring and developing HIV prevention and care continua among transgender women based on the extensive data gathered in the proposed study.

1. Significance:

Strengths

- The proposed study, if successful, would advance knowledge about transgender women's multilevel risks for HIV; about their participation in the prevention continuum and, for those who seroconvert, about their engagement in the care continuum. Information about the wide range of individual, interpersonal, social and structural risk factors would be useful in planning prevention and care and better understanding the lives of the population.
- The proposed study would contribute to increasing estimates of HIV incidence and related factors.

Weaknesses

- The specific innovations in technology for recruitment are not clear. Therefore, it is difficult to assess the potential significance of comparing recruitment methods. Are the methods that are primarily already in use for other health conditions and populations, to be explored in this study, relevant to the transgender women?

2. Investigator(s):

Strengths

- Multiple sites are proposed; investigators at each of the 5 sites have strong records in service delivery and relationships with the population.
- Dr. Wirtz Liu is an early stage investigator with experience working with multiple hidden populations.
- Dr. Reisner also appears to be an early stage investigator with experience working specifically with transgender persons, including testing methods for trans health research as well as conducting studies of determinants of health and the use of health services by the population. He also has experience working directly with the population on translational research.
- Other members of the research team are well experienced with epidemiological research, research and service to transgender women, and technology development.

Weaknesses

- Dr. Wirtz Liu is involved in current funded research focused on transgender women, but these studies have not been completed.

3. Innovation:

Strengths

- Studies are needed that explore how well recruitment methods using geosocial networking (GSN) apps to recruit transgender women. Studies of these methods for recruitment and intervention of related risk groups have been conducted, but their specific relevance to transgender women is less known.
- Comparing technology and nontechnology methods is needed to explore differences in preferences by subgroups.

Weaknesses

- It is not clear if advancements are planned for advancing existing research on the use of GSN and social media or for testing these technologies in their current form on transgender women.
- Several GSN are mentioned as is Facebook for social media; however, additional details about other media like Twitter are not mentioned.

4. Approach:

Strengths

- The application is responsive to the FOA and includes nearly most of the suggested components. The overall plan for each of the aims is clear.
- The investigators propose a comprehensive set of measures at the individual, interpersonal and structural levels.

Weaknesses

- In general, many specifics are not included to help reviewers assess the depth and quality of some of the approaches. For example, there is no table of measurements planned along with their respective validation analysis. Also, the investigators propose to use measurement with known validity and those that are already familiar to the team. They note that they will work to minimize cultural bias and maximize comparability. However, they do not describe the process to adapt these measurements especially those that have not been tested with transgender women.
- Given there are many, many measurements proposed on the participants, it is difficult to know how much burden there will be for the participants.
- What level of readability will be required for all of the measurements? The investigators note that they will use cognitive testing, but they do not describe how they will ensure comprehensibility or validity of response accuracy.
- The role of the Community of Advisory Board (CAB) is not fully described and the CAB could therefore be potentially underutilized. The investigators describe the importance of research “with” the population, but could have done more to describe this concept beyond its core philosophy.
- Specifics about measures of acceptability of the recruitment method are not provided.
- The investigators note that they will not statistically compare efficiency across recruitment methods because of their heterogeneity and that these methods can be classified as technology, event-based or peer referral.

- The letters of support provide an estimated number of transgender women served at the various sites, but the investigators note that they do not plan to recruit TW from HIV care environments. An estimate of the number of potential participants in these five cities that would be eligible if the HIV service recipients are excluded, should be provided.
- A study manual will be produced and training will be provided. However, there is no discussion of how fidelity to these procedures will be assessed and how differences in approaches across, (or even within,) sites will be studied.
- Photos are proposed for the home HIV testing, but it is not clear how the investigators will assure that the online surveys are being completed by the actual participant.

5. Environment:

Strengths

- Each site seems adequately prepared to provide a quality environment.

Weaknesses

- None noted.

CRITIQUE 2

Significance:	4
Investigator(s):	1
Innovation:	2
Approach:	2
Environment:	3

Overall Impact: Given the LITE mandate to recruit highest risk persons in the US who have a disproportionate likelihood of HIV seroconversion, it is wise to access the highest risk individuals. Such a population includes transgender women. The Hopkins-led team intends to recruit and retain such a cohort in five major East Coast urban centers. The East Coast consortium includes leading investigators in the transgender HIV risk field. The investigators' clinical infrastructures and professional backgrounds are well matched to the application. The principal concern is the intensity of the work needed at five sites and whether resources are truly adequate for such ambitious research.

1.Significance

Strengths

- TW are at the highest HIV risk of any population in the U.S.
- Social media may be a good tool for outreach and for maintaining contact.

Weaknesses

- The absolute numbers of TW are small compared to the MSM community, and even a successful program would not move the needle of the overall epidemic very much.

2.Innovation

Strengths

- The phylogeny work can describe the TW networks.

- Social media on a large scale will be the only way to recruit and retain the proposed number of subjects on this modest budget.

Weaknesses

- Given the likelihood of a loss to follow-up, the investigators may consider that a rolling cohort might “age out” the lower risk persons in the cohort. They should permit new enrollment of higher-risk persons.
- There is little attention paid to out-migrants in the application and this factor may result in the loss to follow-up as well.

3.Approach

Strengths

- The outreach strategies are numerous and suitable to recruit TW.
- Only a multi-city approach will secure the numbers of at-risk persons mandated in the RFA.
- The small team of staff at each city will have some support from institutional and allied projects.

Weaknesses

- Phylogeny will only guide the LGBT transmission networks, but will not illuminate much about cross-group transmissions.
- Detailed descriptions of how the target number of TW were chosen per site is not included.

4.Investigators

Strengths

- Dr. Wirtz is a relatively young in her research career, but has the experience to serve as PI.
- The rest of the team includes top TW researchers and experienced health service providers.

Weaknesses

- Network analyses are made possible with or without phylogeny, but there were few details.
- The resources and the field infrastructures may need to be subsidized considerably given modest resources being requested from the NIH.

5.Environment

Strengths

- The five cities chosen make sense for the TW cohort recruitment.
- The letters indicate that there is considerable institutional support for the work.
- While the recruitment numbers are ambitious, the investigators and institutional partners may be able to achieve their goals in this “high risk, high gain” scenario.

Weaknesses

- It is unclear if each site can recruit the numbers as stated.

CRITIQUE 3

Significance:1
Investigator(s):2
Innovation:2
Approach:4
Environment:1

Overall Impact: The proposed study focuses on the significant problem of HIV among transgender women (TW) and on improving outcomes along the HIV prevention continuum (HIVPC) and the HIV continuum of care (HIVCC). Longitudinal cohort studies with diverse transgender women are needed and the present study has the potential to generate data and insights that can have a significant and sustained impact on the field. The overall team is excellent and has a strong record of conducting innovative research with transgender women. The scientific premise of the study is strong and the plan to use home-based HIV testing with photograph validation is innovative. There are some minor concerns that dampen impact. The recruitment, data collection, and retention strategies are contact intensive and the application does not adequately assess for how such contact may affect research outcomes of interest. In addition, the use of the phone app for data collection is not well described. Despite these concerns, the overall impact that the potential study can have on research and clinical practice is significant.

1.Significance:
Strengths

- Transgender women remain one of the population groups that is most heavily impacted by HIV/AIDS. This work will be the largest and most diverse longitudinal cohort study of TW undertaken to date and presents a unique and important opportunity to better understand multi-level factors associated with HIV prevention and engagement in the HIV care continuum for TW.
- Study findings, which will combine self-report and biological markers/phylogenetic data on HIV, STIs, PrEP and hormones, could have a significant impact on subsequent research and care on HIV prevention and treatment.
- The study will yield important insights on recruitment and retention methods for HIV negative and HIV positive TW, which will enhance reproducibility.
- The focus on situated vulnerabilities and the use of biological markers are scientifically sound.

Weaknesses

- None noted.

2. Investigator(s):

Strengths

- The overall Investigative Team is excellent, with all members of the team having relevant and complementary expertise.
- The two PIs have developed a thoughtful multiple PI plan with detailed administrative, technical, and scientific roles for each PI. The governance structure and proposed plan for addressing conflict is appropriate.

Weaknesses

- There is concern that the contact PI is a junior investigator but the overall team and support from senior scientific investigators make this a minor concern.

- There are some inconsistencies in project staffing in the biographical sketches and budget justification. For example, Co-I Poteat's biographical sketch indicates that Dr. Poteat will be a Co-PI but is listed as a Co-I in the application. In the Johns Hopkins budget, a post-doctoral associate is included in the Year 1 budget but the justification states that a post-doctoral associate will be hired and supervised by PI Wirtz in Year 2.

3. Innovation

Strengths

- At-home, rapid HIV-antibody testing with results submitted with photo validation is a highly innovative procedure that has emerging support in the extant literature with men who have sex with men but has not yet been applied to transgender women.
- The study could help to identify hotspots in the HIV prevention and care continuums for transgender women, which could help to develop targeted interventions/services.
- The use of fingerprints to determine identification also is innovative as reliance on identification documents and/or birth certificates is not appropriate for TW.
- The use of computer mediated communication for focus groups is novel and may increase the likelihood of obtaining a diverse sample of TW at each site. Alternative plans are in place for TW who don't have internet access.
- Applying the theory of complex contagion for recruitment is novel.

Weaknesses

- The recruitment methods are not novel per se, but the combination of these methods is necessary in order to generate a large and diverse sample of TW.
- It would help to have an alternative plan for fingerprinting if this method is not acceptable.

4. Approach:

Strengths

- The use of dried blood spots to determine PrEP adherence and use of exogenous hormones are strengths, as are the use of biological markers of viral load/CD4 count and urine samples for STIs testing.
- The application proposes to use blood specimens for phylogenetic analysis.
- Involvement of the Community Advisory Board will further enhance community involvement and relevance of the study.
- The investigators have considered sex as a biological variable and the focus on transgender women is justified.
- Projected estimates of HIV incidence and seroconversion for the go/no go transition plan are appropriate given available data on HIV among TW.
- The approaches are scalable for reaching a large cohort of TW and for meeting the aims articulated in Phase 2 of the study, which will enhance rigor and reproducibility.
- The overall quantitative data analytic plan should produce fairly robust findings on HIV incidence, estimation of risk for factors for HIV seroconversion, and factors associated with HIVPC and HIVCC outcomes, including important biological markers.
- The application has a strong linkage to care plan for participants who may seroconvert during the study.

Weaknesses

- The team provides a compelling rationale for why a LITE approach combined with in-person and other social media strategies are necessary to recruit and retain the sample. At the same time, these strategies are likely to increase the likelihood of a Hawthorne effect. However, the team

should endeavor to categorize and track the different touch points, contacts and services that each participant has to explore the potential influences of such contact over time.
- The scientific rigor would be strengthened by providing a stronger rationale for the data collection schedule. What is gained by having study visits completed every three vs. every six months?
- The use of the home-based app for data collection is not well described. Is there adequate smart-phone use among study participants and will alternative online platforms be made available for participants without a smart phone?
- The recruitment milestones for the go/no-go transition plan would be strengthened by providing an approximate number of how many TW meet eligibility criteria in each study site and how many participants each site will be recruiting. This information is important for understanding recruitment milestones and feasibility.
- A minor concern is that the theories informing the study could be better connected to the stated hypotheses. For example, the concepts of simple contagions, strong ties, and weak ties in the theory of complex contagion are not discussed in the recruitment and analysis sections. Similarly, it is unclear why certain demographic, transition, and structural factors are chosen hypothesis drivers over other factors from the situated vulnerabilities framework.
- What is the option for determining non-duplication of data submission if formative work determines that the proposed fingerprint method is not acceptable?
- Although the qualitative sampling plan is designed to maximize variability, the team may want to focus on a smaller set of factors, because variability across multiple domains/geographical regions will make it difficult to draw meaningful conclusions across subgroups.

5. Environment:

Strengths
- The environment at each institution is excellent with strong systems and supports for the proposed research.
- The inclusion of community-based clinics of Fenway, Callen-Lorde, and Grady Memorial Hospital will facilitate the proposed research.
- The study is further supported by cross-CFAR collaborations at all but one site (Callen-Lorde), which will provide additional support and expertise.

Weaknesses
- None noted

CRITIQUE 4

Significance:	1
Investigator(s):	1
Innovation:	2
Approach:	4
Environment:	1

Overall Impact: This application has a high level of significance, strong investigators, and excellent environments. Collecting a large sample of only transgender women and studying them longitudinally within five metropolitan areas of the eastern and southern US is highly significant, but the argument presented for innovation is weak and seems more of an argument for significance, addressing what will be accomplished rather than why accomplishing this work is innovative. The approach has many

important strengths, but also some important weaknesses especially an imprecise go/no go transition milestone.

1. Significance:

Strengths

- Following a large sample of only transgender women for 24 months, using the combination of technology-based and non-technology-based recruitment and retention.
- Including a large diverse subsample.
- Identification of risk factors for HIV seroconversion, access to HIV prevention, and linkage to care for those who HIV seroconvert, which can inform evidence-based HIV prevention interventions.
- Ability to identify potential microepidemics.

Weaknesses

- None noted.

2. Investigator(s):

Strengths

- The application is led by a very strong team of investigators with extensive related research experience studying transgender women from the five metropolitan areas covered by the study as well as established relationships with local community-based organizations.

Weaknesses

- None noted.

3. Innovation:

Strengths

- The application will collect a large sample of transgender women and studying them longitudinally within five metropolitan areas of the eastern and southern US.

Weaknesses

- The argument provided for innovation in the application is more of an argument for significance addressing what will be accomplished rather than why it is innovative.

4. Approach:

Strengths

- Basing the UG3 phase work on social network communications theories and the Theory of Contagion and the UH3 phase work on a situated vulnerabilities framework.
- The application will use a combination of technology- and non-technology-based recruitment and retention methods.
- Baseline comparisons of HIV uninfected and infected groups are included. For instance, the baseline HIV uninfected group will be followed longitudinally at 3 month intervals for up to 24

months and HIV seroconverters at 3 month intervals for 6 months after seroconversion to assess engagement in the HIV care continuum.

- Working with a Community Advisory Board that is comprised of transgender community leaders and those serving in transgender communities is a strength.
- The use of on-line focus groups to inform recruitment, marketing, and app development.
- Dried blood spots for participants will be collected for reporting pre-exposure prophylaxis (PrEP) or exogenous hormone use.
- The UG3 phase will assess the efficiency of alternate recruitment methods, baseline comparison of HIV uninfected and infected participants, and exploration of baseline profiles using latent class analysis.
- Rotating on-line and facility-based data collection with surveys rotating in length and format but always including the same core set of measures is proposed.
- A thorough quality assurance/quality control plan is in place.
- The use of the scan statistic for detecting seroconversion event clusters in time and space.

Weaknesses

- Hypothesis 1 addresses differences in technology- and non-technology-based recruitment methods compared to non-technology based methods alone. These do not seem to be two distinct groups; the second one seems to be a subset of the first.
- The go/no go transition milestone seems imprecise. It states that the HIV seroconversion rate is anticipated to be 4/100py. It does not state that the go decision requires a specific seroconversion rate.
- The go/no go transition milestone calls for recruitment of 1,100 participants, but the analytic plan proposed to track attrition to guarantee enrollment of only 1,000 participants.
- The research strategy does not provide a plan for sharing data and resources with other LITE projects and so it does not address the secondary objective of the RFA which is to facilitate interactions and sharing among awardees.

5. Environment:

Strengths

- The five partner institutions have extensive access to transgender women, experience recruiting and retaining them, with four of the five having the extra benefit of CFAR resources.

Weaknesses

- None noted.

THE FOLLOWING RESUME SECTIONS WERE PREPARED BY THE SCIENTIFIC REVIEW OFFICER TO SUMMARIZE THE OUTCOME OF DISCUSSIONS OF THE REVIEW COMMITTEE ON THE FOLLOWING ISSUES:

PROTECTION OF HUMAN SUBJECTS (Resume): ACCEPTABLE (CODE 30)
Data and Safety Monitoring Plan/Board: ACCEPTABLE

INCLUSION OF WOMEN: ACCEPTABLE (CODE 2A)

INCLUSION OF MINORITIES: ACCEPTABLE (CODE 1A)

INCLUSION OF CHILDREN: NOT APPLICABLE (CODE 3A)

BIOHAZARDS COMMENT: NOT APPLICABLE

FOREIGN INSTITUTION: NOT APPLICABLE

SELECT AGENTS: NOT APPLICABLE

DATA SHARING PLANS: UNACCEPTABLE

Comments: Biologic specimens do not appear to be retained for sharing. The application includes a dissemination plan and will take advantage of the newly created Data Management Services and Data Conservancy at Johns Hopkins, an open access, HIPAA compliant data repository that is operated in accordance to NSF guidelines. The Resource Plan states that the team will follow NIH public access policy but does not reference the LITE consortium or address how the data will be shared with other LITE projects.

MODEL ORGANISM SHARING PLANS: NOT APPLICABLE

GENOMIC DATA SHARING PLAN: NOT APPLICABLE

AUTHENTICATION OF KEY BIOLOGICAL AND/OR CHEMICAL RESOURCES: ACCEPTABLE

BUDGETARY OVERLAP: NOT APPLICABLE

COMMITTEE BUDGET RECOMMENDATIONS: ACCEPTABLE

SCIENTIFIC REVIEW OFFICER'S ADMINISTRATIVE NOTES: Clarification is necessary to describe if the hiring of a post-doctoral fellow under Dr. Wirtz' supervision will take place in year one of the project.

Footnotes for 1 UG3 AI133669-01; PI Name: Wirtz, Andrea

NIH has modified its policy regarding the receipt of resubmissions (amended applications). See Guide Notice NOT-OD-14-074 at http://grants.nih.gov/grants/guide/notice-files/NOT-OD-14-074.html. The impact/priority score is calculated after discussion of an application by averaging the overall scores (1-9) given by all voting reviewers on the committee and multiplying by 10. The criterion scores are submitted prior to the meeting by the individual reviewers assigned to an application, and are not discussed specifically at the review meeting or calculated into the overall impact score. Some applications also receive a percentile ranking. For details on the review process, see http://grants.nih.gov/grants/peer_review_process.htm#scoring.

MEETING ROSTER
National Institute of Allergy and Infectious Diseases Special Emphasis Panel

NATIONAL INSTITUTE OF ALLERGY AND INFECTIOUS DISEASES
Limited Interaction Targeted Epidemiology (LITE) to Advance HIV Prevention (UG3/UH3)
ZAI1 AL-A (M1)
04/19/2017 - 04/20/2017

Notice of NIH Policy to All Applicants: Meeting rosters are provided for information purposes only. Applicant investigators and institutional officials must not communicate directly with study section members about an application before or after the review. Failure to observe this policy will create a serious breach of integrity in the peer review process, and may lead to actions outlined in NOT-OD-14-073 at https://grants.nih.gov/grants/guide/notice-files/NOT-OD-14-073.html and NOT-OD-15-106 at https://grants.nih.gov/grants/guide/notice-files/NOT-OD-15-106.html, including removal of the application from immediate review.

CHAIRPERSON(S)

RUTLEDGE, SCOTT EDWARD, PHD
ASSOCIATE DEAN, ASSOCIATE PROFESSOR
COLLEGE OF PUBLIC HEALTH
TEMPLE UNIVERSITY
PHILADELPHIA, PA 19122

SILVERBERG, MICHAEL J, PHD, MPH
RESEARCH SCIENTIST III
DIVISION OF RESEARCH
KAISER PERMANENTE NORTHERN CA
OAKLAND, CA 94612

MEMBERS

AMIRKHANIAN, YURI A, PHD
PROFESSOR
DEPARTMENT OF PSYCHIATRY AND BEHAVIORAL MEDICINE
CENTER FOR AIDS INTERVENTION RESEARCH
MEDICAL COLLEGE OF WISCONSIN
MILWAUKEE, WI 53202

ANDRASIK, MICHELE PEAKE, PHD
SENIOR STAFF SCIENTIST/AFFILIATE CLINICAL PROFESSOR
VACCINE AND INFECTIOUS DISEASE DIVISION
DEPARTMENT OF GLOBAL HEALTH
FRED HUTCHINSON/UNIVERSITY OF WASHINGTON
SEATTLE , WA 98105

BOURIS, ALIDA M, PHD
ASSOCIATE PROFESSOR
SCHOOL OF SOCIAL SERVICE ADMINISTRATION
UNIVERSITY OF CHICAGO
CHICAGO, IL 60637

CHAN, PHILIP ANDREW, MD, MS
ASSISTANT PROFESSOR
DEPARTMENT OF MEDICINE
BROWN UNIVERSITY
PROVIDENCE, RI 02906

CHEN, XINGUANG, MD, PHD
PROFESSOR
DEPARTMENT OF EPIDEMIOLOGY
UNIVERSITY OF FLORIDA
GAINSVILLE, FL 32610

HALL, IRENE, PHD, MPH
DEPUTY DIRECTOR FOR SURVEILLANCE, EPIDEMIOLOGY AND LABORATORY SCIENCE
DIVISION OF HIV/AIDS PREVENTION (DHAP)
CENTERS FOR DISEASE CONTROL AND PREVENTION (CDC)
ATLANTA, GA 30329

KNAFL, GEORGE J, PHD
PROFESSOR
DEPARTMENT OF FAMILY HEALTH/NURSING
UNIVERSITY OF NORTH CAROLINA AT CHAPEL HILL
CHAPEL HILL, NC 27599

MCFARLAND, WILLIAM, MD, PHD, MPH
PROFESSOR
DEPARTMENT OF EPIDEMIOLOGY AND BIOSTATISTICS
UNIVERSITY OF CALIFORNIA, SAN FRANCISCO
SAN FRANCISCO, CA 94102-6033

NOVITSKY, VLADIMIR A, PHD, MD
SENIOR RESEARCH SCIENTIST
DEPARTMENT OF IMMUNOLOGY AND INFECTIOUS DISEASES
HARVARD SCHOOL OF PUBLIC HEALTH
BOSTON, MA 02115

POND, SERGEI L KOSAKOVSKY, PHD
PROFESSOR
DEPARTMENT OF BIOLOGY/IGEM
TEMPLE UNIVERSITY
PHILADELPHIA, PA 19122

QIAN, HAN-ZHU, MB, PHD
HEALTH SCIENTIST
DIVISION OF GLOBAL HIV & TB
CENTER FOR DISEASE CONTROL
ATLANTA, GA 30329

SCHNALL, REBECCA, PHD, MPH, RN
MARY DICKEY LINDSAY ASSISTANT, PROFESSOR OF
DISEASE PREVENTION AND HEALTH PROMOTION
SCHOOL OF NURSING
COLUMBIA UNIVERSITY
NEW YORK, NY 10032

VERMUND, STEN H, MD, PHD
PROFESSOR/DEAN
YALE SCHOOL OF PUBLIC HEALTH
YALE UNIVERSITY
NEW HAVEN, CT 06520

WRAY, TYLER BLAKE, PHD
ASSISTANT PROFESSOR (RESEARCH)
DEPARTMENT OF BEHAVIORAL AND SOCIAL SCIENCES
SCHOOL OF PUBLIC HEALTH
BROWN UNIVERSITY
PROIVIDENCE , RI 02912

SCIENTIFIC REVIEW OFFICER

LAU, AUDREY OI-TING, PHD
SCIENTIFIC REVIEW OFFICER
SCIENTIFIC REVIEW PROGRAM
DIVISION OF EXTRAMURAL ACTIVITIES
NATIONAL INSTITUTES OF HEALTH/NIAID
ROCKVILLE, MD 20892

EXTRAMURAL SUPPORT ASSISTANT

POINTER, LARISA Y.
EXTRAMURAL SUPPORT ASSISTANT
SCIENTIFIC REVIEW PROGRAM
DIVISION OF EXTRAMURAL ACTIVITIES
NATIONAL INSTITUTES OF HEALTH/, NIAID
ROCKVILLE, MD 20852

PROGRAM REPRESENTATIVE

LEE, SONIA S, PHD
PROGRAM OFFICER
MATERAL AND PEDIATRIC INFECTIOUS DISEASE BRANCH
NATIONAL INSTITUTES OF HEALTH/NICHD
ROCKVILLE, MD 20892

SHARP, GERALD B., DRPH
PROGRAM OFFICER
EPIDEMIOLOGY BRANCH
DIVISION OF AIDS
NATIONAL INSTITUTES OF HEALTH/NIAID
ROCKVILLE, MD 20852-9830

STIRRAT, MICHAEL J, PHD
PROGRAM OFFICER
HIV TREATMENT AND TRANSLATIONAL SCIENCE BRANCH
NATIONAL INSTITUTES OF HEALTH/NIMH
ROCKVILLE, MD 20892

WILLIAMS, CAROLYN F., PHD
CHIEF, EPIDEMIOLOGY BRANCH
EPIDEMIOLOGY BRANCH
DIVISION OF AIDS
NATIONAL INSTITUTES OF HEALTH/NIAID
ROCKVILLE, MD 20852-9830

ZIMUND, LORI B BSC
HEALTH SPECIALIST
BASIC SCIENCE PROGRAM
NATIONAL INSTITUTES OF HEALTH/ NIAID
ROCKVILLE, MD 20892

GRANTS MANAGEMENT REPRESENTATIVE

RODRIGUEZ, CYNTHIA M.
GRANTS MANAGMENT SPECIALIST
GRANTS MANAGEMENT PROGRAM
DIVISION OF EXTRAMURAL ACTIVITIES
NATIONAL INSTITUTES OF HEALTH/NIAID
ROCKVILLE, MD 20892

Consultants are required to absent themselves from the room during the review of any application if their presence would constitute or appear to constitute a conflict of interest.
